# Effect of enamel protective agents on shear bond strength of orthodontic brackets

**DOI:** 10.1186/s40510-014-0034-0

**Published:** 2014-07-18

**Authors:** Mona A Montasser, Mahasen Taha

**Affiliations:** Department of Orthodontics, Faculty of Dentistry, Mansoura University, Mansoura, 35516 Egypt

**Keywords:** Enamel protective agents, Shear bond strength, ARI scores

## Abstract

**Background:**

This paper aimed to study the effect of two enamel protective agents on the shear bond strength (SBS) of orthodontic brackets bonded with conventional and self-etching primer (SEP) adhesive systems.

**Methods:**

The two protective agents used were resin infiltrate (ICON) and Clinpro; the two adhesive systems used were self-etching primer system (Transbond Plus Self Etching Primer + Transbond XT adhesive) and a conventional adhesive system (37% phosphoric acid etch + Transbond XT primer + Transbond XT adhesive ). Sixty premolars divided into three major groups and six subgroups were included. The shear bond strength was tested 72 h after bracket bonding. Adhesive remnant index scores (ARI) were assessed. Statistical analysis consisted of a one-way ANOVA for the SBS and Kruskal-Wallis test followed by Mann-Whitney test for the ARI scores.

**Results:**

In the control group, the mean SBS when using the conventional adhesive was 21.1 ± 7.5 MPa while when using SEP was 20.2 ± 4.0 MPa. When ICON was used with the conventional adhesive system, the SBS was 20.2 ± 5.6 MPa while with SEP was 17.6 ± 4.1 MPa. When Clinpro was used with the conventional adhesive system, the SBS was 24.3 ± 7.6 MPa while with SEP was 11.2 ± 3.5 MPa. Significant differences in the shear bond strength of the different groups (*P* = .000) was found as well as in the ARI scores distribution (*P* = .000).

**Conclusion:**

The type of the adhesive system used to bond the orthodontic brackets, either conventional or self-etching primer, influenced the SBS, while the enamel protective material influenced the adhesive remnant on the enamel surface after debonding.

## Background

Enamel demineralization and white spot lesions associated with orthodontic fixed appliances is one of the greatest challenges faced by clinicians at the end of the orthodontic treatment not only for esthetic reasons but also because this subsurface demineralization represents the first stage of caries formation [1-4].

Different methods have been studied, all aiming to reduce enamel demineralization during orthodontic treatment without compromising the bond strength of the orthodontic brackets. The most common method was the use of fluoride-containing mouth rinses, gels, and tooth pastes [5-7]; however, studies found a significant association between the patient compliance to the rinsing program advised by the clinician and the reduction in the development of white spot lesions [8]. It was found that with only standardized general prophylactic measures, new white spot lesions developing on the maxillary front teeth during orthodontic treatment were seen in 60.9% of the patients [9].

Preventive measures that do not depend on the patient’s compliance have been developed and gained popularity to solve the problem of demineralization. These included the use of glass ionomer cement [10,11], topical applications of preventive agents as fluoride and casein phosphopeptide-amorphous calcium phosphate [12,13], antibacterial agents incorporated in the adhesive resin [14,15], fluoride releasing adhesives [16,17], caries infiltration resins [18,19], laser irradiation [20,21], bioactive glass-containing adhesives [22], and enamel deproteinizing agents [23].

The current study focused on two preventive agents Clinpro and ICON. Clinpro is a fluoridated varnish containing 5% sodium fluoride. Fluoride was found to be effective in reducing the development of white spot lesions associated with fixed orthodontic treatment [16,24]. Also, ICON resin infiltration was found to decrease the dissolution of enamel and so limit the appearance of white spot lesions [25]. When is the proper timing to apply these materials to get the best result of decreasing the white spot lesions around the orthodontic brackets is a worthwhile question. These preventive agents could be applied after bonding the orthodontic brackets, but this may not be easy all the times especially where there are severely crowded or partially erupted teeth. The other option is to apply these materials before bonding the orthodontic brackets, but these preventive agents could have an effect on the shear bond strength and/or the amount of adhesive left on the teeth after debonding of the orthodontic brackets upon treatment completion.

The objective of this study was to study the effect of using the two enamel protective agents before bonding on the shear bond strength of orthodontic brackets bonded with conventional and self-etch adhesive systems.

## Methods

This *in vitro* testing used 60 extracted human upper premolars stored in an aqueous solution of thymol (0.1% wt/vol). Teeth were extracted as part of orthodontic treatment and collected to be used in research. To calculate the sample size, Epicalc 2000 software version 1.02 (Brixton Books, Brixton, UK) was used. The sample size was found to be ten specimens for each group based on 80% power and 95% confidence interval.

**Figure 1:**
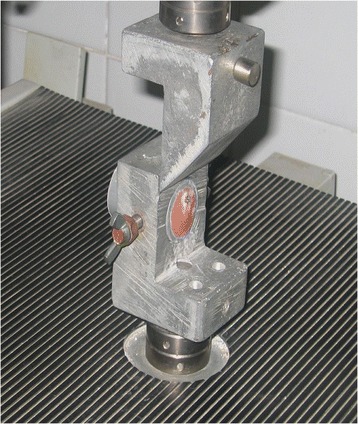
**Typically** **mounted specimen for SBS testing.**

The teeth were fixed in self-curing acrylic resin placed in flexible molds with the roots embedded in the acrylic and the crown exposed and oriented perpendicularly to the bottom of the mold.

Two types of enamel protective agents were used in the current study: ICON (DMG, Hamburg, Germany) and Clinpro (3M Unitek, Monrovia, CA, USA). The two adhesive systems used in this study were Transbond XT light cure adhesive and Transbond Plus Self Etching Primer (3M Unitek, Monrovia, CA, USA), and Transbond XT light cure adhesive, Transbond XT primer, and 37% phosphoric acid (3M Unitek, Monrovia, CA, USA). All materials were used according to the manufacturers’ instructions.

The sample was divided into three major groups; group 1 used (ICON) before bonding the orthodontic brackets, group 2 used Clinpro before bonding, and group 3 a control group with no protective enamel agent used. Each group was divided into two subgroups; in the first one, orthodontic brackets were bonded with self-etching adhesive system and in the second one, a conventional adhesive system was used.

Premolar stainless steel brackets (Equilibrium 2 Roth prescription, 0.022 in. slot size, Dentaurum Orthodontics, Ispringen, Germany) were used. The buccal surface of each tooth was cleaned with non-fluoride oil-free pumice paste using a nylon brush attached to a slow-speed hand piece for 5 s, and then the tooth was rinsed with water for 10 s and dried with an oil-free air spray. Brackets were bonded to the teeth according to the manufacturer’s instructions for the adhesive system and stored in distilled water at 37°C until testing.

Bracket debonding was performed 72 h after bonding in a material testing unit (model no 5500, Instron Corp, Canton, MA, USA) with an occluso-gingival load applied to the bracket base. The shearing rod was adjusted each time so the shearing blade is parallel to the base of the bracket contacting it in a reproducible way each test. The shear force was applied to the bracket by lowering the shearing rod perpendicularly in the gingival direction, producing a shear force at the bracket-enamel interface (Figure 1).

The crosshead speed was 2.0 mm/min, and the failure load in Newton was divided by the bracket base bonding area of 10.90 mm^2^ to calculate the shear bonding strength in MPa.

The adhesive remnant index (ARI) and failure site assessment was completed immediately after each shear bond strength debonding under ×20 magnification [26]. The ARI evaluation used the 4-point scale of Årtun and Bergland [27] where 0 indicates no adhesive left on the tooth surface, implying that bond fracture occurred at the resin/enamel interface; 1 indicates less than half the resin left on the tooth surface, implying that bond fracture occurred predominantly at the resin/enamel interface; 2 indicates more than half the resin left on the tooth surface, implying that bond fracture occurred predominantly at the bracket/resin interface; and 3 indicates all resin left on the tooth surface, with a distinct impression of the bracket base, implying that bond fracture occurred at the bracket/resin interface.

Descriptive statistics, including mean, standard deviation, and minimum and maximum values of the shear bond strength, were calculated for each of the adhesive systems tested. Analysis of variance (ANOVA) test followed by a LSD *post hoc* multimeans comparison test was used to compare the groups. A Kruskal-Wallis test was used in conjunction with a Mann-Whitney test to compare the differences in the ARI scores between the groups. Significance for all statistical tests was at *P* ≤ .05. Statistics were carried out using Statistical Package for Social Sciences (SPSS Inc, Chicago, IL, USA) program version 10.

## Results

The descriptive statistics of the SBS of each group are presented in Table 1. The one-way ANOVA, Table 1, indicated significant differences in the shear bond strength of the different groups (*P* = .000). When using Clinpro before bonding with SEP and Transbond XT, the SBS was significantly less than the other groups; the two control groups, the conventional adhesive group (*P* = .000) and the SEP group (*P* = .001); the two ICON groups, the conventional adhesive (*P* = .001) and the SEP group (*P* = .015); and Clinpro with the conventional adhesive system (*P* = .000). When using Clinpro before bonding with the conventional adhesive, the bond strength was similar to that of the other groups but significantly higher than the SBS when using ICON before bonding with SEP and Transbond XT adhesive.

**Table 1:** **Descriptive statistics of the** ***in vitro*** **shear bond strength (MPa)**

	**Number**	**Mean**	**SD**	**Minimum**	**Maximum**
Transbond XT + ICON + H_3_PO_4_^a,d^	10	20.2	±4.6	11.0	28.6
Transbond XT + ICON + SEP^bd^	10	17.6	±4.1	13.3	23.7
Transbond XT + Clinpro + H_3_PO_4_^a^	10	21.3	±6.6	13.4	34.2
Transbond XT + Clinpro + SEP^c^	10	11.2	±3.5	5.6	15.0
Transbond XT + primer + H_3_PO_4_^a,d^	10	21.1	±7.5	11.2	34.7
Transbond XT + SEP^a,d^	10	20.2	±4.0	14.0	25.9

*F* = 6.07; *P* = .000. Mean values in each row with the same letter are not significantly different at *P* ≤ .05.

The results of the Kruskal-Wallis test showed that the ARI scores, Table 2, were significantly different (*P* = .000) between the groups. The Mann-Whitney test showed no difference in the ARI scores between self-etching and conventional etching groups when using ICON (*P* = .166), Clinpro (*P* = .802), as well as when bonding to untreated enamel in the control group (*P* = .751). Results of one-way ANOVA showed also that the type of preventive agent used on the enamel significantly influenced the ARI scores distribution; there was a significant difference depending on whether it was ICON or Clinpro that was used before bonding with SEP (*P* = .005) or with phosphoric acid etching (*P* = .000).

**Table 2:** **Freq** **uencies of the ARI scores for the two groups**

	**Number**		**ARI scores**		
		**0**	**1**	**2**	**3**
Transbond XT + ICON + H_3_PO_4_^a^	10	0	1	1	8
Transbond XT + ICON + SEP^a^	10	0	3	2	5
Transbond XT + Clinpro + H_3_PO_4_^b^	10	5	3	2	0
Transbond XT + Clinpro + SEP^b^	10	6	2	1	1
Transbond XT + primer + H_3_PO_4_^c^	10	2	4	2	2
Transbond XT + SEP^c^	10	3	4	0	3

Chi-square =22.77; *P* = .000; 0 indicates no adhesive left on the tooth surface, 1 indicates less than half the resin left on the tooth surface, 2 indicates more than half the resin left on the tooth surface, and 3 indicates all resin left on the tooth surface, with a distinct impression of the bracket base. Scores in each row with the same letter are not significantly different at *P* ≤ .05.

## Discussion

The lowest SBS was recorded with the samples treated with Clinpro before bonding the orthodontic brackets; the SBS in this group was significantly lower than the SBS in the other five groups. This could be attributed to the resistance effect that the outer enamel layer acquires from the fluoride content of the Clinpro which may be of significant effect especially when using self-etching primers in bonding due to their more superficial etching effect compared with the etching of the conventionally used phosphoric acid. Previous studies [28-30] with scanning electron microscope (SEM) indicated that although self-etch priming agents have the potential to etch the enamel surface, the etching pattern is less deep compared to the etching pattern of phosphoric acid. A chemical bonding capacity through the interaction between some functional monomers and the calcium of residual hydroxyapatite may contribute favorably to the bonding effectiveness [31-33], but fluoride affects the enamel surface rendering it more resistant to demineralization. Fluoride in low concentrations favors the formation of fluoro-hydroxyapatite, which is less susceptible to acidic solubility than hydroxyapatite [34,35]. Therefore, it is recommended to use these preventive agents after bonding the brackets when self-etch adhesive systems are used.

In the current study, using the caries infiltrant (ICON) before bonding did not significantly change the bond strength compared to the other groups, although the bond strength was lower when self-etching primer was used than when phosphoric acid was used for enamel preparation before bonding. This was also observed in the control group; shear bond strength was lower when self-etching primer was used than when phosphoric acid was used, but this difference was statistically insignificant. Previous studies found a significant increase in the shear bond strength of Transbond XT adhesive with phosphoric acid and Transbond XT primer when ICON was used before bonding orthodontic brackets to sound enamel [36] or even to demineralized enamel [37]. The shear bond strength was also increased when Transbond Plus Self Etching Primer was used instead of the conventional phosphoric acid etching to sound enamel [36]. The shear bond strengths recorded in this study were sufficient for clinical use in all the six groups presenting different combinations of adhesive systems and enamel protective agents as well as control groups. The average range of bond strength was suggested by Reynolds [38] to be 5.9 to 7.8 MPa for clinical and 4.9 MPa for laboratory performances. *In vitro* and *in vivo* studies of SBS are both needed; *in vitro* measurements of shear bond strength provide useful information about the bonding efficiency of different types of materials, but the actual performance of these materials can only be evaluated in the environment where they were intended to function [39]. Unfortunately, no one variable or combination of variables that can be measured in the laboratory is perfectly predictive of what might occur when the bonding adhesive is used in the demanding environment of the oral cavity [40-42]. Therefore; *in vitro* studies are mainly important as a preliminary guide to the clinician, while *in vivo* studies are needed for evidence-based practice.

The distribution of the ARI scores was assessed in this study under ×20 magnifications [26]. Although different quantitative and qualitative methods have been used to assess the ARI scores after orthodontic bracket debonding and quantitative methods were found preferable if accurate evaluation of the adhesive remnant is required [43], ARI score evaluation system has proved to be of value in the studies of orthodontic adhesive systems. ARI score system is a quick and simple method that needs no special equipment. Although SEM evaluation might be more accurate than evaluation under ×10 or ×20 magnification, it is harder to be reflected in clinical applications [26]. The distribution of the ARI scores was found different between the three major groups. In the ICON group, for both self-etching and conventional etching subgroups, higher ARI scores tended to be more frequent, while in Clinpro and control groups, both self-etching and conventional etching subgroups, less adhesive remnant tended to be seen left on the enamel surface after debonding. This could be attributed to the chemical bond between the resin infiltrant and the adhesive resin. However, the adhesive remaining on the enamel surface after debonding was not different in the three major groups between the self-etching subgroup and the conventional etching subgroup indicating a similar effect of the enamel protective material with the two types of adhesive systems. These results differed from the results of Naidu et al. [36] study that found using ICON as preconditioning before bonding orthodontic brackets to sound enamel did not affect ARI scores distribution compared to the control groups using Transbond XT primer and Transbond PSEP. The importance of the site of bond failure was found not to be a reflection of bond strength; therefore, the site of failure did not reflect different bond strengths at different interfaces [44,45]. On the other hand, a variety of factors could affect bond strength including the type of enamel conditioner, acid concentration, length of etching time, composition of the adhesive, bracket base design, bracket material, oral environment, skill of the clinician, and time of light exposure in case of light-cure approach [46].

Applying the results of this study clinically, it would be preferred using Clinpro after bonding the orthodontic brackets when self-etch adhesive systems are used, while it could be used before bonding when conventional adhesive systems are used. ICON resin infiltrate, on the other hand, could be used before bonding with either of the two adhesive systems, but removal of large amount of adhesive remnant would be needed.

## Conclusions

Based on the above findings, we conclude the following:

1. Overall, the SBS was lower when self-etching primer was used than when phosphoric acid was used for enamel preparation before bonding in the three major groups.
2. Significantly lower SBS was recorded when Clinpro was used before bonding using the self-etching adhesive system.
3. The ICON group showed the higher ARI scores to be more frequent, while Clinpro and control groups showed lower ARI scores more frequently. The adhesive remnant was not different between the self-etching and the conventional etching subgroups.
